# Targeting chemoattractant chemokine (C–C motif) ligand 2 derived from astrocytes is a promising therapeutic approach in the treatment of neuromyelitis optica spectrum disorders

**DOI:** 10.3389/fimmu.2023.1144532

**Published:** 2023-03-28

**Authors:** Yupeng Wang, Jiangping Bian, Mengyuan Yao, Li Du, Yun Xu, Haoxiao Chang, Hengri Cong, Yuzhen Wei, Wangshu Xu, Huabing Wang, Xinghu Zhang, Xingchao Geng, Linlin Yin

**Affiliations:** ^1^ Department of Neuroinfection and Neuroimmunology, Beijing Tiantan Hospital, Capital Medical University, Beijing, China; ^2^ China National Clinical Research Center for Neurological Diseases, Beijing, China; ^3^ National Center for Safety Evaluation of Drugs, National Institutes for Food and Drug Control, Beijing, China; ^4^ Chinese Academy of Medical Sciences and Peking Union Medical College, Beijing, China

**Keywords:** neuromyelitis optica spectrum disorders (NMOSD), chemokine (C–C motif) ligand 2 (CCL2), aquaporin 4 antibodies (AQP4-IgG, NMO-IgG), astrocytes, inflammatory signal pathway

## Abstract

**Introduction:**

Aquaporin-4 immunoglobulin G (AQP4-IgG)-induced astrocytes injury is a key mechanism in the pathogenesis of neuromyelitis spectrum disorder (NMOSD), and although CCL2 is involved, its specific role has not been reported. We aimed to further investigate the role and potential mechanisms of CCL2 in AQP4-IgG-induced astrocyte injury.

**Methods:**

First, we evaluated CCL2 levels in paired samples of subject patients by automated microfluidic platform, Ella®. Second, we knock down astrocyte's CCL2 gene in vitro and in vivo to define the function of CCL2 in AQP4-IgG-induced astrocyte injury. Third, astrocyte injury and brain injury in live mice were assessed by immunofluorescence staining and 7.0T MRI, respectively. Western blotting and high-content screening were conducted to clarify the activation of inflammatory signaling pathways, and changes in CCL2 mRNA and cytokine/chemokines were measured by qPCR technique and flow cytometry, respectively.

**Results:**

There were greatly higher CSF-CCL2 levels in NMOSD patients than that in other non-inflammatory neurological diseases (OND) groups. Blocking astrocyte CCL2 gene expression can efficiently mitigate AQP4-IgG-induced damage *in vitro* and *in vivo*. Interestingly, prevention of CCL2 expression could decrease other inflammatory cytokines released, including IL-6 and IL-1β. Our data suggest that CCL2 involves in the initiation and plays a pivotal role in AQP4-IgG-damaged astrocytes.

**Discussion:**

Our results indicate that CCL2 may serve as a promising candidate target for inflammatory disorder therapy, including NMOSD.

## 1 Introduction

Neuromyelitis optica spectrum disorder (NMOSD) is a primary astrocytopathic disease associated with inflammation of the central nervous system (CNS), demyelination, and tissue injury. The disease is characterized by, often severe, optic neuritis and transverse longitudinally extensive myelitis, leading to blindness or paralysis in most cases (1–3). A serum autoantibody biomarker, neuromyelitis optica (NMO)-immunoglobin G (IgG), targeting aquaporin 4 (AQP4) water channels, which is enriched in the foot processes of astrocytes, has been identified in the central pathogenesis of NMOSD. Despite the known interaction of NMO-IgG with AQP4, the mechanisms responsible for the development of NMO lesions remain unknown.

The degree of the NMO lesion depends on astrocyte exposure and response to environmental factors in the CNS. Binding of NMO-IgG to AQP4 triggers numerous immune-associated pathways, including interferon, chemokine/cytokine, complement, and NF-κB signaling pathways, which can result in the recruitment of innate immune cells into the CNS, and the concomitant amplification and exacerbation of tissue dyshomeostasis and injury (4–6). The chemokine (C–C motif) ligand 2 (CCL2), also known as monocyte chemotactic protein-1 (MCP-1), is a potent C–C chemokine receptor type 2 (CCR2)-positive myeloid cell chemoattractant and plays an important role in the progression of various CNS diseases associated with the infiltration of myeloid cells (7, 8). Peripheral monocytes are known to enter the brain after traumatic brain injuries and seizures and contribute to neuronal injury and axonal damage (8, 9). Pathological features of NMO autopsies show reactive macrophage/microglia accumulated in a focal pattern in the pial, ependymal, and other AQP4 immunoreactivity regions in the brain (10). CD14^+^CD16^++^ non-classical monocytes, which are CCR2 negative (CCR2^–^), are up-regulated in the peripheral blood of patients with NMO (11). Based on previous observations, we hypothesized that binding of NMO-IgG to AQP4 triggers an increase in CCL2 in astrocytes, and that CCL2 up-regulation subsequently causes the inflammatory cascade that is the underlying pathogenic event in NMOSDs.

To test this hypothesis, first, we compared the levels of CCL2 in serum and cerebrospinal fluid (CSF) of paired samples from NMOSD patients with patients with other non-inflammatory neurological diseases (ONDs). Second, we investigated the function of CCL2 in NMO-IgG-induced pathogenicity models *in vivo* and *in vitro*. Our understanding of the fate of CCL2 in NMOSD pathogenesis may help us to develop novel therapeutic strategies.

## 2 Methods

### 2.1 Patients and samples

Paired serum and cerebrospinal fluid (CSF) samples were collected from NMOSD patients and OND patients in the Department of Neurological Infection Immunology, Beijing Tiantan Hospital, affiliated with Capital Medical University, from August 2017 to March 2021. All the samples were stored at –80°C until use. In the present study, the diagnosis of NMOSD was based on the revised 2015 NMOSD criteria. The OND group included patients with primary headache (*n* = 4), benign intracranial hypertension (*n* = 2), and outer nerve palsy (*n *= 1). The study was approved by the Ethics Committee of Beijing Tiantan Hospital, affiliated with the Capital Medical University, Beijing, China (No. AF/02-06.01/07.0). Written informed consent was obtained from all patients.

### 2.2 Detection of chemokine ligand 2 of paired samples

Human CCL2/MCP-1 levels were measured using the Ella^®^ automated microfluidic platform. According to the manufacturer’s instructions, the Protein Simple Plex™ kit (ProteinSimple, SPCKB-PS-001108) was used to measure the content of CCL2 in the paired serum and CSF samples. In brief, the samples had to be removed from the refrigerator, thawed, heated, and then diluted in a 1:1 ratio with the sample buffer solution, shaken, thoroughly mixed, and centrifuged to eliminate any visible particles before being added to the assay apparatus (ProteinSimple).

### 2.3 Primary astrocytes culture

As previously described (5), 1-day-old Wistar rats were purchased from Beijing Vital River Laboratory Animal Technologies Co. Ltd. (Beijing, China), and the cerebral cortices were taken for mixed glial cultures. The mixed glial cells were filtered through a 70-μm cell strainer and plated on poly-L-lysine-coated culture plates, and then cultured in Dulbecco’s modified Eagle medium (DMEM) (Gibco™), which was supplemented with 10% heat-inactivated fetal bovine serum (Gibco) and 1% penicillin/streptomycin (Gibco). The plates were incubated at 37°C in a humidified atmosphere of 5% CO_2_ and 95% air. After about 12 days, microglia, endothelial cells, and oligodendrocyte cells were separated from the mixed glial cells by shaking *in vitro*. The astrocytes were then purified from primary mixed glial cell cultures by two trypsin digestions. Glial fibrillary acidic protein (GFAP)-positive cells accounted for more than 95% of the cultured cells.

### 2.4 NMO-IgG purification

NMO-IgG was isolated from surplus clinical serum specimens that were sterile-filtered serum pools (i.e., AQP4-IgG seropositive NMOSD) using HiTrap Protein G HP (GE Healthcare, Bio-Sciences, Piscataway, NJ, USA) as previously described (5). To summarize, serum samples were diluted in a 1 : 1 ratio in binding buffer. Samples were applied to the columns after the columns were filled with binding buffer. The columns were then washed again with binding buffer, and the antibodies were eluted in accordance with the manufacturer’s instructions. Finally, the antibodies were concentrated using Amicon Ultra-4 centrifugation units with 10,000 MW cut-off points (Merck Millipore, Billerica, MA, USA). AQP4 Autoantibody ELISA Version 2 Kit (AQP4/96/2, RSR) was used to measure the concentration of AQP4-IgG in the samples, which was 58.3 units/mL. The concentrated IgG was then sterile filtered at 0.22 μm and stored at –80°C.

Control-IgG was a product from Jackson ImmunoResearch Ltd. Co., USA (cat. no. 009-000-002). Astrocytes pretreated with Control-IgG served as negative controls.

### 2.5 siRNA transfection in astrocytes

Primary astrocytes at a density of 1 × 10^5^ cells per well were seeded into a 24-well plate and cultured overnight in DMEM (#11965-092; Gibco) without antibiotics and fetal bovine serum. Then, in accordance with the manufacturer’s protocol, siRNA was transfected into cells with a Lipo3000 kit (Invitrogen, USA). The dosages of Lipo3000 were as follows: Lipo3000 : siRNA = 1.0 (μL) : 2.5 (μL). CCL2 siRNA: *forward* 5′-GCAUCAACCCUAAGGACUUTT-3′, *reverse* 5′-AAGUCCUUAGGGUUGAUGATT-3′, negative control siRNA: *forward* 5′- UUCUCCGAACGUGUCACGUTT-3′, *reverse* 5′-ACGUGACACGUUCGGAGAATT-3′.

A total of 48 hours later, the transfected cells were harvested and processed for other experimental treatments, such as RT-qPCR, Western blotting, cellular immunostaining, cell viability evaluation, and cytokine/chemokine measurement.

### 2.6 RNA isolation and qPCR

As previously described (5), the total RNA was isolated from primary rat astrocytes or brain tissues of mice using TRIzol™ reagent (Invitrogen). A NanoDrop^®^ ND-1000 spectrophotometer (NanoDrop Technologies, Wilmington, DE, USA) was used to determine RNA purity and concentration. RNA (1 μg) was used to synthesize cDNA with the Roche Transcriptor First Strand cDNA Synthesis Kit (Roche Holding AG, Basel, Switzerland) using anchored oligo (dT) and random hexamer primers. The relative *CCL2* mRNA level was determined using SYBR™ green-based qPCR, which was normalized with glyceraldehyde-3-phosphate dehydrogenase (GAPDH) or actin. The tat *CCL2* primers were: *forward* 5*′*-AGGTCTCTGTCACGCTTCTG-3′ and *reverse* 5′-GTTCTCCAGCCGACTCATTG-3′. The mouse *CCL2* primers were: *forward* 5′- TTCCACAACCACCTCAAGCA-3′ and *reverse* 5*′*- TTAAGGCATCACAGTCCGAGTC-3′. The GAPDH primers were: *forward* 5′-AAGTTCAACGGCACAGTCAAG and *reverse* 5′-ACATACTCAGCACCAGCATCA-3′. The actin primers were: *forward* 5′- GCAAGTGCTTCTAGGCGGAC-3’′ and *reverse* 5′-AAGAAAGGGTGTAAAACGCAGC-3′. Data were analyzed using the 2^–ΔΔCt^ method.

### 2.7 Western blotting

First, we used the RIPA buffer containing protease and phosphatase inhibitors to lyze the treated cells. Second, we extracted targeted proteins using the Nuc-Cyto-Mem Preparation Kit (#P1201; APPLYGEN, China). A total of 30 μg of protein was separated using 10% sodium dodecyl sulfate—polyacrylamide gel electrophoresis (SDS-PAGE) and then transferred to a polyvinylidene difluoride (PVDF) membrane. We blocked the membrane with 5% bovine serum albumin (BSA) before incubating the primary antibodies overnight at 4°C. The membrane was incubated with the horseradish peroxidase-labeled secondary antibodies for 1 hour at room temperature after being washed three times with TBST (tris-buffered saline with Tween^®^). The following proteins’ expressions were determined using the Western lighting technique: AQP4 (#ABN411, 1 : 1,000; Sigma-Aldrich), p38 mitogen-activated protein kinase (MAPK) (#8690; 1 : 1,000; CST), p-p38 MAPK (#4511; 1 : 1,000; CST), nuclear factor kappa B (NF-κB) (#6956,;1 : 1,000; CST), p-NF-κB (#3033, 1 : 1,000; CST), β-actin (#TA-09; 1 : 1,000; ZSGB-BIO), β-tubulin (#C1340; 1 : 1,000; APPLYGEN), and ATPase (#CY5159; 1 : 1,000; Abways). A semi-quantitative statistic was used to indicate the relative expression of the target protein, i.e., target protein grayscale value/internal reference protein grayscale value. The software Genesys (GBOX-CHEMI-XX9-E; Syngene, UK), which quantifies proteins, was used to count the grayscale values of each protein.

### 2.8 Cell immunostaining and imaging

The cells were seeded evenly in 24-well plates with 7 × 10^4^ cells per well. After treatments, the cells were washed with cold phosphate-buffered saline (PBS), fixed with 4% paraformaldehyde for 15 minutes, washed with PBS, and infiltrated with a 3% BSA solution containing 0.3% Triton X-100 (#T824275; Mecklin, Shanghai, China) for 1 hour. Cells were then co-incubated with the following antibodies: anti-GFAP (#130300; 1 : 300; Invitrogen), anti-AQP4 (#ABN411; 1 : 300; Sigma-Aldrich, USA), anti-phosphor-p38 (#4511s; 1 : 200; CST), or anti-phosphor-NF-κB p65 (#3033s; 1 : 200; CST) overnight at 4°C in a humidity chamber. The next day, the cells were washed and incubated for 1 hour at room temperature with goat anti-rat IgG/Cy3 (#bs-0923G-Cy3; 1 : 400; Bioss) to label GFAP and Alexa Fluor 488-conjugated goat anti-rabbit IgG (#A11034; 1 : 400; Invitrogen) to label AQP4, phosphor-p38, and phosphor-NF-κB p65. Finally, the coverslips were rinsed in PBS and mounted in a 4′,6-diamidino-2-phenylindole (DAPI)-containing mounting medium (#ZLI-9557; ZSGB-BIO, Beijing, China). Images were acquired using an LSM780 inverted confocal microscope (Carl Zeiss). All images were captured under the same conditions within a given experiment, and 3–5 independent experiments were carried out. The average fluorescence intensity was analyzed using ZEN software (version 2.3).

### 2.9 High-content screening

Primary astrocytes were seeded into a 96-well plate at a density of 6 × 10^3^ cells per well. After treatments, the cells were washed with cold PBS, fixed with 4% paraformaldehyde for 15 minutes, washed with PBS, and then infiltrated with a 3% BSA solution containing 0.3% Triton X-100 for 1 hour. Cells were then incubated overnight at 4°C in a humidity chamber with the following antibodies: anti-GFAP (#130300; 1 : 300; Invitrogen), anti-phosphor-Janus kinase 1 (JAK1) (#74129s; 1 : 200; CST), anti-phosphor-JAK2 (#3776s; 1 : 200; CST), anti-phosphor-p38 (#4511s; 1 : 200; CST), anti-phosphor p44/42 (#4370s; 1 : 200; CST), anti-phosphor-AKT (#4060s; 1 : 200; CST), and anti-phosphor-NF-κB p65 antibody (#3033s; 1 : 200; CST). Cells were then washed and incubated for 1 hour with goat anti-rat IgG/Cy3 (#bs-0923G-Cy3; 1 : 400; Bioss) to label GFAP and Alexa Fluor 488-conjugated goat anti-rabbit IgG (#A11034; 1 : 400; Invitrogen) to label other antibodies. Cells were imaged and analyzed using a high-content analyzer (ArrayScan XTI; Thermo Fisher Scientific).

### 2.10 Cell viability assay

The primary astrocytes were seeded into 96-well plates coated with poly-L-lysine at a density of 1.3 × 10^4^ cells per well, and then treated with CON-IgG or NMO-IgG for 4 hours. Subsequently, 10 μL of CCK-8 solution (#CK04, DOJINDO, Japan) was added to each well and the plates were incubated for 1 hour. The optical density values were measured at 450 nm. Cell survival rate = (optical density value of experimental group – optical density value of blank group)/(optical density value of control group – optical density value of blank group) × 100%.

### 2.11 Cytokine/chemokine measurement *in vitro*

The supernatants from each astrocyte group treated by different conditions were collected and analyzed with the LEGEND plex Rat Inflammation Panel (#740401; BioLegend, USA). The culture supernatant assay was carried out per the LEGEND plex protocol, and all samples were analyzed by CytoFLEX flow cytometry.

### 2.12 Animals and NMO-IgG intracerebral injection

Twenty female C57BL/6 mice were purchased from Beijing Vital River Laboratory Animal Technology Co. Ltd. and divided into vehicle/sham, vehicle/NMO-IgG, and adeno-associated virus (AAV)-CCL2 shRNA/NMO-IgG subgroups. The AAV [pAAV-GfaABC1D-EGFP-3Xflag-miR30shRNA (CCL2)-Woodchuck hepatitis virus post-transcriptional regulatory element (WPRE)] containing an astrocyte-specific promoter was injected into the tail vein of mice in the AAV-CCL2 shRNA/NMO-IgG group (*n* = 7), which can significantly reduce the *CCL2* gene expression in astrocytes. pAAV-GfaABC1D-EGFP-3Xflag-miR30shRNA (NC)-WPRE AAV was injected into the tail vein of mice in the vehicle/NMO-IgG group (*n* = 7) as an interfering-free control. Mice in the vehicle/sham group received 0.9% NaCl solution (*n* =2). Obio Technology Crop Ltd. (Shanghai, China) was responsible for the virus’s design, packaging, and verification. Mice were housed in cages, and exposed to a 12-hour light–dark cycle at 18–23°C and 38%–42% humidity, with free access to food and water. All mice were weighed and monitored daily, and all experiments were carried out on age-matched adult female mice (8–12 weeks old). Procedures used in this protocol were performed in accordance with the National Institutes of Health Guidelines for the Care and Use of Animals and were approved by the Ethics Committee of Beijing Tiantan Hospital, affiliated with the Capital Medical University.

Three weeks (21 days) after mice received vehicle or AAVs *via* tail vein injection, NMO-IgG was administered in the mice’s brain parenchyma. Briefly, mice were anesthetized by an intraperitoneal injection of 10% chloral hydrate (0.04 mL/kg) and then mounted in a stereotaxic frame. The bregma and posterior fontanelle were exposed *via* a midline scalp incision, and a burr hole was made 2 mm to the right of the bregma. A gas-tight glass syringe with a linked 26-gauge needle was implanted 3 mm deep to deliver 20 μL of NMO-IgG to the parenchymal tissue at a rate of 1 μL/min. In the vehicle/sham group, 20 μL of a 0.9% normal saline solution was injected into the parenchyma tissue. The rectal temperature was maintained at 37°C throughout the process using a heating lamp.

### 2.13 7.0-T MRI neuroimaging

In this study, lesion volumes were evaluated in live mice on day 3 after NMO-IgG injection using 7.0-T small-animal MRI. Briefly, mice were anesthetized by an intraperitoneal injection of tribromoethanol, and during the scanning procedure, the normal body temperature (36–37°C) of mice was maintained by a heated circulating water blanket.

T2-weighted images were acquired with follows parameters to detect lesion: TR = 3,080 ms; effective TE = 41 ms. A total of 17 slices were acquired with thickness = 0.5 mm; field of view = 2.4 cm × 3.0 cm; and matrix 192 × 320. The MRI data were analyzed using ImageJ software.

### 2.14 Tissue immunostaining and imaging

On day 24, mice were executed after MRI scanning, and the brain was removed for the immunostaining procedure. Briefly, mice were transcardially perfused with 0.9% NaCl and 4% paraformaldehyde (PFA) after being given 0.04 mL/kg of 10% chloral hydrate to induce profound anesthesia. Subsequently, we removed the brains and post-fixed them in 4% PFA at 4°C overnight. Then, the brain was cut into coronal slices (20 μm) using a frozen slicer (CM 1950; Leica, Bensheim, Germany). Then, the floating slices were washed three times with PBSTT (phosphate-buffered saline [PBS], with 0.3% Triton X-100 and 0.1% Tween) and blocked with 1% goat serum to prevent the binding of non-specific antibodies, and then incubated at 4°C overnight with antibodies against GFAP (#130300, 1:200; Invitrogen) and AQP4 (#ABN411; 1 : 200; Sigma-Aldrich). Slices were incubated with goat anti-rat IgG/Cy3 (#bs-0923G-Cy3; 1 : 400; Bioss) and goat anti-rabbit IgG (#A11034 1 : 400; Invitrogen) the following day at room temperature for 1 hour after being washed with PBS. Finally, all slices were incubated with DAPI. Images were captured using the PE Multispectral Tissue Intelligent Imaging Analysis System (PENEOIMAGER, USA). The data were quantified using ImageJ software.

### 2.15 Statistical analysis

One-way ANOVA was used to examine the statistical differences in the measurements between two or more groups. Welch’s corrections were used after the unpaired *t*-test if the variances were significantly different. The Shapiro–Wilk test was used to determine normality. If the data did not conform to a normal distribution, the Mann–Whitney *U*-test was used. Count data were compared using the chi-squared test. Experimental results were expressed as mean ± standard error (SE). Data were plotted and analyzed using GraphPad Prism (version 8.0; GraphPad Software Inc., CA, USA) and IBM SPSS Statistics version 26.0 (IBM Corporation, Armonk, NY, USA). A *p*-value < 0.05 was considered a statistically significant difference.

## 3 Results

### 3.1 Clinical demographics

Paired serum and CSF samples were collected from 59 NMOSD patients and seven OND patients. The clinical characteristics of all subjects are presented in **Table 1**. There were no significant differences in the sex ratio, age, disease duration, or white blood cell count in the CSF between the two groups. The seropositivity ratio for anti-AQP4 antibodies among NMOSD patients was 33 out of 59 (55.9%). The QAlb and CSF protein levels were significantly higher in NMOSD patients than in OND patients. The QAlb represents the CSF-to-serum albumin ratio. As albumin cannot be synthesized in the CNS and the blood–brain barrier (BBB) limits its crossover from the peripheral to the central nervous system, the QAlb can be used to measure BBB permeability. Our data suggest that the degree of BBB disruption is more severe in NMOSD patients than in OND patients.

**Table 1:** Demographic data and clinical characteristics of patients.

	NMOSD (*n* = 59)	OND (*n* = 7)	*p*-value
Gender (female/male)	40/19	3/4	0.19
Age (years), mean (SD)	43.3 ± 15.3	44.7 ± 12.2	0.94
Disease duration (months), median (range), months	16.1 (0–168)	9.8 (2–49)	0.37
EDSS at onset, median (range)	4 (0–9)	–	–
Seropositivity for anti-aquaporin 4 antibody, *n* (%)	33 (55.9)	–	–
QAlb (× 10^–3^), median (range)	4.9 (1.1–107.4)	3.5 (2.5–6.0)	0.04*
CSF WBC (count/μL), median (range)	6.0 (0–1371)	2.0 (1–7)	0.18
CSF protein level (mg/dL), median (range)	32.5 (10.6–581.5)	27.1 (22.5–34.3)	0.04*

Continuous variables are shown as the mean ± SD, non-continuous variables are shown as the median (range), and categorical variables are described as numbers (percentages). SD, standard deviation; EDSS, Expanded Disability Status Scale; QAlb, albumin quotient, presented as CSF-to-serum albumin ratio; WBC, white blood cell. *p < 0.05.

### 3.2 Evaluation of CCL2 levels in paired samples

**Figure 1:**
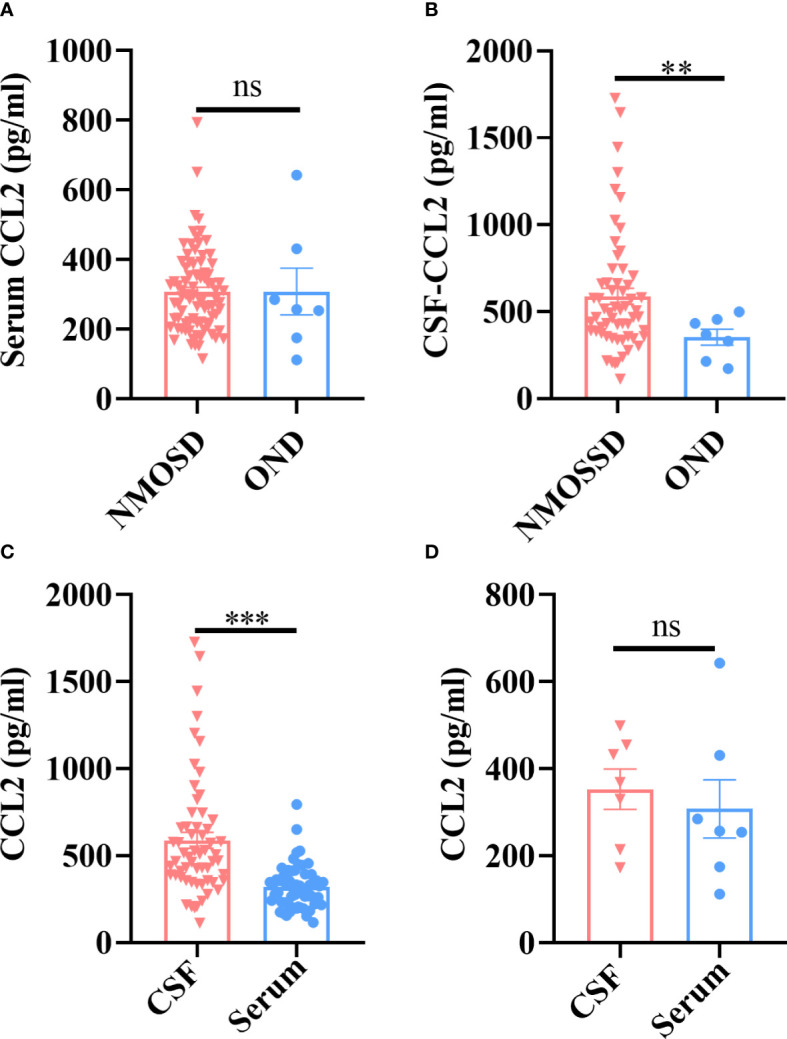
Patients with NMOSD and OND had their CCL2 levels in their serum and CSF tested by the Ella^®^ device (Protein Simple). Paired serum and CSF samples were available from 59 patients with NMOSD and seven patients with OND. **(A)** There were no significant differences in the serum CCL2 levels between the NMOSD and OND groups. **(B)** CCL2 levels in the CSF were noticeably higher in individuals with NMOSD than in patients in the OND group. **(C)** In NMOSD patients, CCL2 levels were significantly higher in CSF than in matched serum samples. **(D)** In OND patients, there were no significant differences in the levels of CCL2 between the matched serum and CSF samples. Data are presented as the mean ± SE. **p < 0.01; ***p < 0.001; ns, not significant (*p* > 0.05).

First, we detected the levels of CCL2 in all subjects by comparing paired serum and CSF samples. Our results revealed no significant differences in serum CCL2 levels between NMOSD and OND patients (*p *> 0.05) (**Figure 1A**); however, levels of CCL2 in CSF were significantly higher in NMOSD patients than in OND patients (*p *< 0.01) (**Figure 1B**). Moreover, CSF-CCL2 levels were significantly higher than their paired serum samples in NMOSD patients (*p *< 0.001) (**Figure 1C**), whereas no significant differences were found in CCL2 levels between paired CSF and serum samples in OND patients (*p *> 0.05) (**Figure 1D**).

### 3.3 Discriminating value of CCL2 levels in AQP4-IgG seropositive and seronegative NMOSD patients

**Figure 2:**
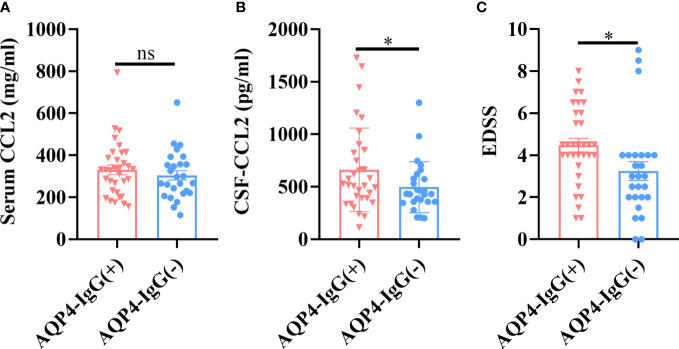
CCL2 levels in different NMOSD subgroups. AQP4-IgG-seropositive (AQP4-IgG+) (*n* = 33) and AQP4-IgG-seronegative (AQP4-IgG–) (*n* = 26) subsets were further separated from the total NMOSD samples (*n* = 59). **(A)** There were no significant differences in serum CCL2 levels between the two groups. **(B)** CSF CCL2 levels in the AQP4-IgG-seropositive group were significantly higher than in the AQP4-IgG-seronegative group. **(C)** Patients with AQP4-IgG-seropositive NMOSD had considerably higher EDSS scores than those with seronegative NMOSD. Data are presented as the mean ± SE. *p < 0.05, ns, not significant (*p* > 0.05).

There were no significant differences in CCL2 levels from serum samples in NMOSD patients with AQP4-IgG seropositive and seronegative samples (*p *> 0.05) (**Figure 2A**); however, CSF-CCL2 levels were significantly higher in AQP4-IgG-seropositive patients than in AQP4-IgG-seronegative patients (*p *< 0.05) (**Figure 2B**). AQP4-IgG-seropositive patients had significantly higher Expanded Disability Status Scale (EDSS) scores than AQP4-IgG-seronegative patients (*p *< 0.05) (**Figure 2C**). Our results suggest that AQP4-IgG induces a significant CCL2 release in the CSF of NMOSD patients. Therefore, CCL2 may play a key role in AQP4-IgG-induced pathogenesis.

### 3.4 NMO-IgG exposure induced significant release of CCL2 from astrocytes *in vitro*

As we know, the astrocyte end-foot is a critical component of the BBB. The AQP4 protein expressed on the astrocytic end-foot is a target of NMO-IgG. Previous studies have reported that NMO-IgG (mainly AQP4 antibodies) binds to its target protein, AQP4, and subsequently triggers internalization, which causes astrocyte injury (12). To determine the exact role of CCL2 in the damaging effect of NMO-IgG on astrocytes, an astrocytes model was established *in vitro*, and different experimental conditions were evaluated. Rat primary astrocytes were exposed to NMO-IgG at different concentrations, or were co-incubated with NMO-IgG at a fixed concentration for different periods of time. Finally, we choose an NMO-IgG concentration of 4 units (U) and an incubation period of 4 hours as the ideal conditions for further study (**Supplementary Figure 1**).

**Figure 3:**
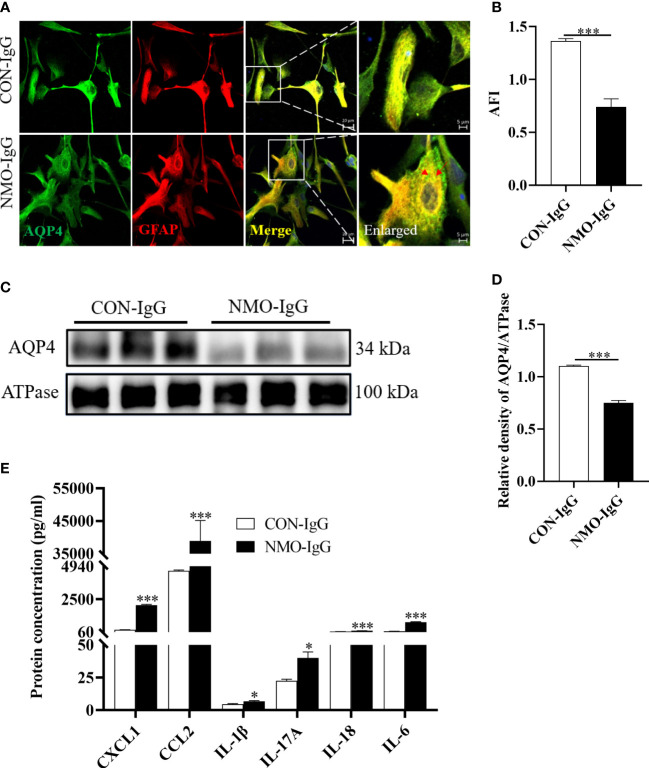
Damage effects of NMO-IgG on astrocytes *in vitro*. Rat primary astrocytes were exposed to either NMO-IgG (4 U) purified from a serum pool of more than 10 AQP4-IgG-seropositive NMOSD patients or human commercial control IgG (CON-IgG) for 4 hours. **(A)** The AQP4 protein (green) was uniformly distributed on the cytomembrane of the astrocytes after CON-IgG incubation, whereas, after NMO-IgG incubation, there were obvious AQP4 protein aggregations on the cytomembrane and some of these were internalized into the cytoplasm and degraded into granules. The scale bars in merged and enlarged pictures are 20 and 5 μm, respectively. **(B)** Compared with the CON-IgG group, the average fluorescence intensity (AFI) of AQP4 in the NMO-IgG group significantly decreased. **(C)** Cytomembrane lysates from astrocytes were immunoblotted with antibodies against AQP4, and NMO-IgG induced a significant decrease in AQP4 expression on cell membranes compared with the CON-IgG group. **(D)** The ratio of AQP4 density to its internal reference, ATPase, is shown. **(E)** NMO-IgG caused the release of several chemokines or cytokines, with CCL2 being the most pronounced. Data are presented as the mean ± SE of three or five independent experiments. ***p < 0.001. The red arrows indicate areas of AQP4 protein aggregation, symbol "*" indicates p < 0.05.

As shown in **Figure 3**, after incubation of astrocytes with NMO-IgG (4 U) for 4 hours, the expression of AQP4 (green) and GFAP (red) in astrocytes was detected by cellular immunofluorescence staining. The amount of AQP4 expressed on the cytomembrane of astrocytes was also measured by Western blotting, and the content of CCL2 in the cell supernatant was determined by flow cytometry. The data revealed that, after incubation of astrocytes with CON-IgG, the AQP4 was uniformly distributed on the cytomembrane of astrocytes, whereas NMO-IgG induced the aggregation of AQP4 on the cytomembrane of astrocytes into granules and internalization to the cytoplasm (**Figure 3A**). The average fluorescence intensity (AFI) of AQP4 was significantly lower in the NMO-IgG group than in the CON-IgG group (*p *< 0.001) (**Figure 3B**). NMO-IgG can induce a marked decrease in the expression of AQP4 protein on the cytomembrane of astrocytes (*p *< 0.001) (**Figures 3C, D**). Compared with CON-IgG, NMO-IgG induces the significant release of several chemokines and cytokines, including CCL2, CXCL1, IL-1β, IL-17A, IL-18, and IL-6 (**Figure 3E**) (*p* < 0.05). In the present study, CCL2 showed the sharpest increase out of all the cytokines detected (*p* < 0.001). Together, our results indicate that CCL2 may be involved in NMO-IgG-induced damage in astrocytes.

### 3.5 CCL2 silence effectively reduces NMO-IgG damage to astrocytes

**Figure 4:**
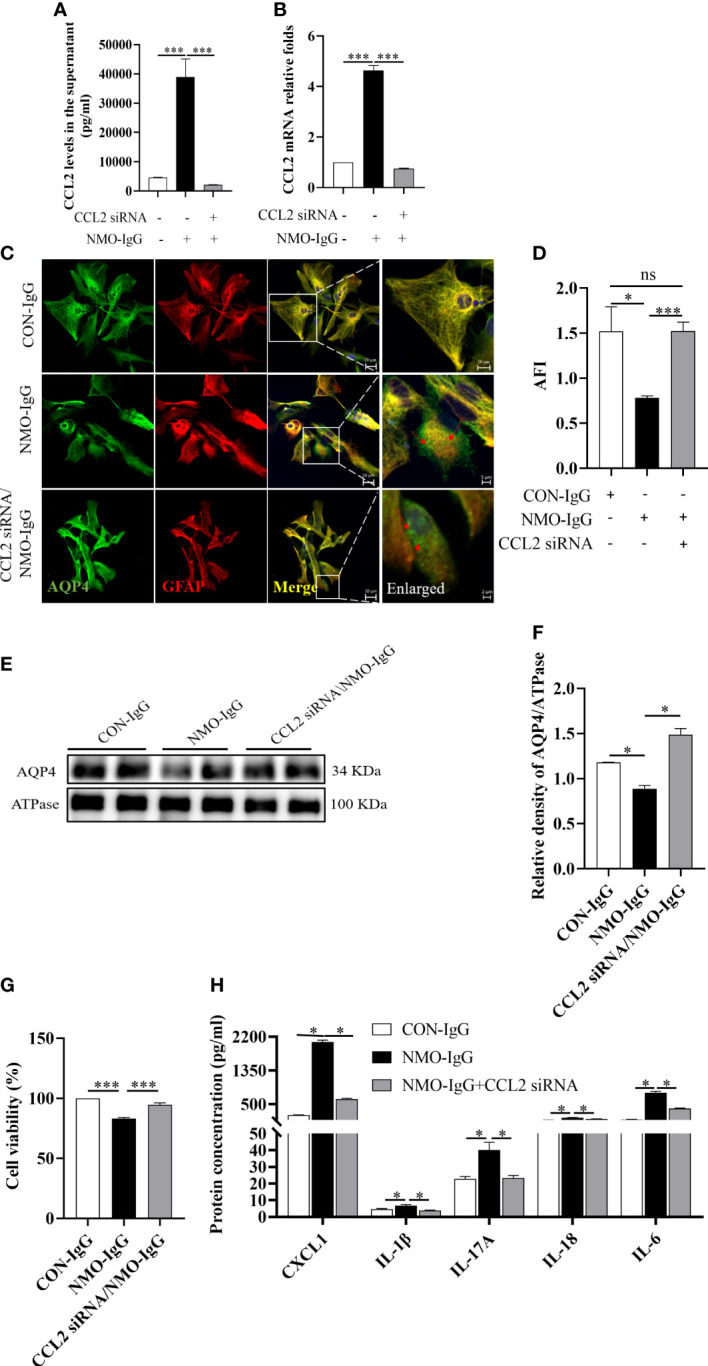
The effect of CCL2 siRNA on NMO-IgG-induced astrocyte damage. Interfering with CCL2 siRNA dramatically inhibited the release of CCL2 soluble proteins into the supernatant **(A)** and the expression of CCL2 mRNA **(B)** produced by NMO-IgG incubation. **(C)** Following interference with CCL2 expression by pretreatment with CCL2 siRNA, the degree of AQP4 aggregation into granules of the cell membrane or internalization into the cytoplasm was markedly reduced in the NMO-IgG-treated group. The scale bars in merged and enlarged pictures are 20 and 5 μm, respectively. **(D)** Compared with the NMO-IgG group, CCL2 siRNA significantly prevented the NMO-IgG-induced drop in AFI. **(E)** The protein expression of AQP4 on the cytomembrane was significantly lower in the NMO-IgG group than in the CON-IgG group, whereas there was no significant drop in the CCL2 siRNA/NMO-IgG group. **(F)** The ratio of AQP4 optical density to its internal reference, ATPase, is shown. **(G)** A high concentration of NMO-IgG (8 U) severely reduced cell viability in rat primary astrocytes. Impressively, the damage caused by NMO-IgG at the high concentration could be reversed following CCL2 interference. **(H)** Interfering with CCL2 siRNA significantly reduced the release of various additional inflammatory factors, including CXCL1, IL-1, IL-17A, IL-18, and IL-6. Data are presented as the mean ± SE of three or five independent experiments. *p < 0.05, ***p < 0.001; ns, not significant (*p* > 0.05). The red arrows indicate areas of AQP4 protein aggregation.

To investigate the role of CCL2 in NMO-IgG-injured astrocytes, we used CCL2 small interfering RNA (siRNA) to silence CCL2 expressions. Then NMO-IgG was incubated with rat primary astrocytes that either had or had not received CCL2 siRNA; CON-IgG incubation served as a negative control. CCL2 released into the supernatant and mRNA expressions were measured to verify the efficiency of RNA interference (**Figures 4A, B**). By using immunofluorescence staining, we discovered that CCL2 siRNA effectively prevented NMO-IgG-induced astrocyte damage, including preventing the accumulation of AQP4 in granules in the astrocyte cytosol and internalization to the cytoplasm as well as the reduction of AQP4 expressions (*p* < 0.001) (**Figures 4C, D**). The results obtained from the immunoblot assay were consistent with the findings of morphological staining (**Figures 4E, F**).

A high concentration (8 U) of NMO-IgG can induce a significant decrease in cell viability in normal astrocytes (*p* < 0.001) (**Figure 4G**); however, the lethal effect of NMO-IgG was significantly reversed after CCL2 siRNA interference (*p* < 0.001). Moreover, CCL2 siRNA could significantly inhibit the production of CXCL1, IL-1β, IL-17A, IL-18, and IL-6 by NMO-IgG stimulation and significantly improves the cellular inflammatory status (**Figure 4H**).

### 3.6 Inhibition of CCL2 expression derived from astrocytes reversed NMO-IgG damage in mice

**Figure 5:**
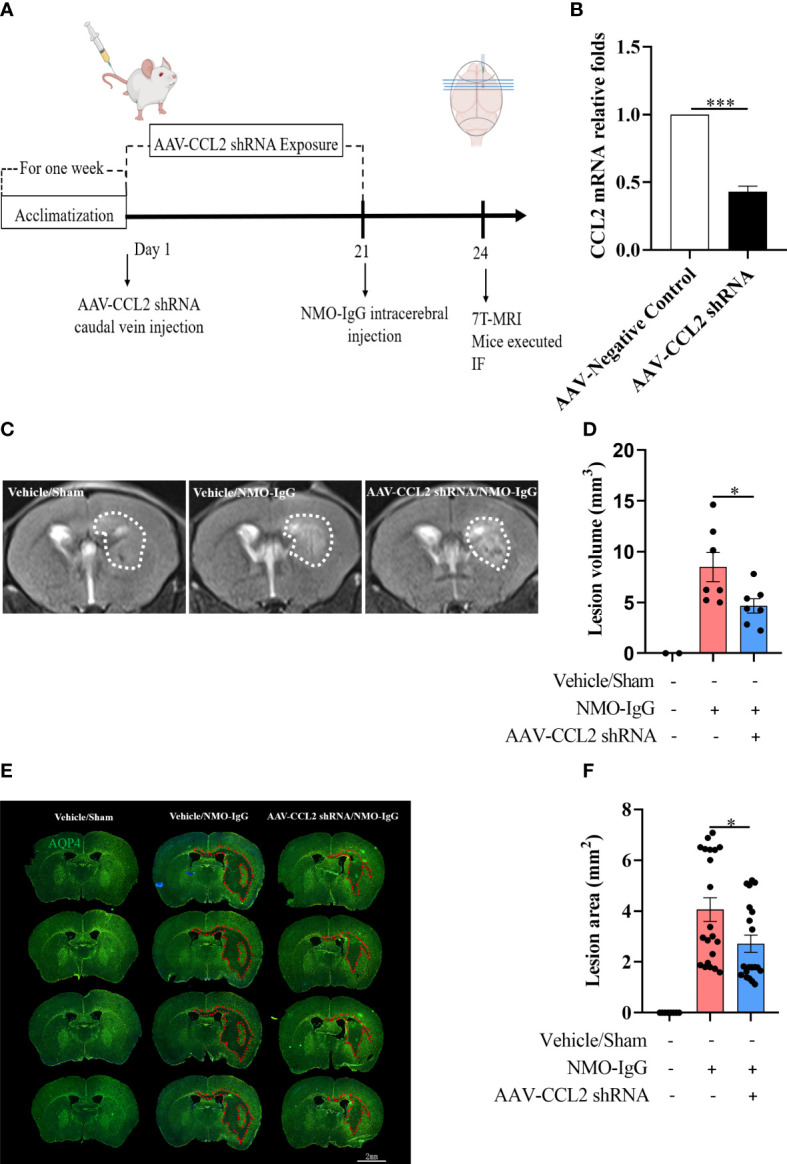
Selective knockdown of *CCL2* gene expression in astrocytes by AAV decreased brain focus lesions and AQP4 loss. **(A)** The schematic shows the experimental design. C57BL/6 mice received AAV (pAAV-GfaABC1D-EGFP-3Xflag-miR30shRNA (CCL2)-WPRE) containing an astrocyte-specific promoter by injection into the tail vein on day 1 (n = 7 per group), which can significantly reduce the expression of the *CCL2* gene in brain astrocytes. Mice received vehicle or NMO-IgG (intracerebral injection) on day 21. At the indicated time points after NMO-IgG administration, mice were subjected to 7.0-T MRI and immunofluorescence staining. **(B)** Efficiently reduces the CCL2 mRNA expression in astrocytes by AAV-CCL2 shRNA injection. **(C)** On day 24, mice underwent 7.0-T MRI, and T2-weighted images showed lesion areas dotted with white dashed lines. **(D)** Quantification of the lesion volume induced by NMO-IgG after receiving AAV-CCL2 shRNA or not. Compared with the vehicle/NMO-IgG group, the lesion volume in the AAV-CCL2 shRNA/NMO-IgG group reduced significantly. **(E)** Immunostaining of AQP4 in NMO lesions on day 24. Red dash lines delineate the lesion area. Scale bar = 2 mm. **(F)** Quantification of the lesion area with AQP4 loss in the sections of vehicle/NMO-IgG and AAV-CCL2 shRNA/NMO-IgG group mice. Data are given as mean ± SE. *p < 0.05, ***p < 0.001.

In the *in vivo* model, we selectively knocked down the *CCL2* gene in astrocytes in the brains of mice using an adenovirus-containing GFAP promoter and CCL2 shRNA 21 days before NMO-IgG damage (**Figures 5A, B**). Animals from vehicle/NMO-IgG and vehicle/sham groups received a 0.9% NaCl solution, and the astrocytic CCL2 expression was normal. We then induced a NMO animal model through the intracerebral injection of NMO-IgG in mice. Mice in the vehicle/sham group received the same procedure as those in the sham control group. On day 24, 7.0-T MRI scanning was performed to visualize lesions (T2) and calculate the lesion volume. We found a significant reduction in lesion volume in AAV-CCL2 shRNA-pretreated NMO mice compared with the vehicle/NMO-IgG group (**Figures 5C, D**). In addition, NMO lesion sizes were evaluated using immunofluorescence staining for AQP4 and GFAP (GFAP not shown). In the vehicle/sham group of mice, reactive astrocytes proliferated noticeably at the injection site without any AQP4 loss. Vehicle/NMO-IgG group mice displayed a significant loss of AQP4. Compared with the vehicle/NMO-IgG group, AAV-CCL2 shRNA-pretreated NMO mice lost less AQP4 at the injection site (**Figures 5E, F**). Our results suggest that astrocytic CCL2 prevention effectively reduces NMO-IgG-induced damage *in vitro* and *in vivo*.

### 3.7 MAPK p38 and NF-κB signaling pathway activation involved in CCL2 increase induced by NMO-IgG stimulation

**Figure 6:**
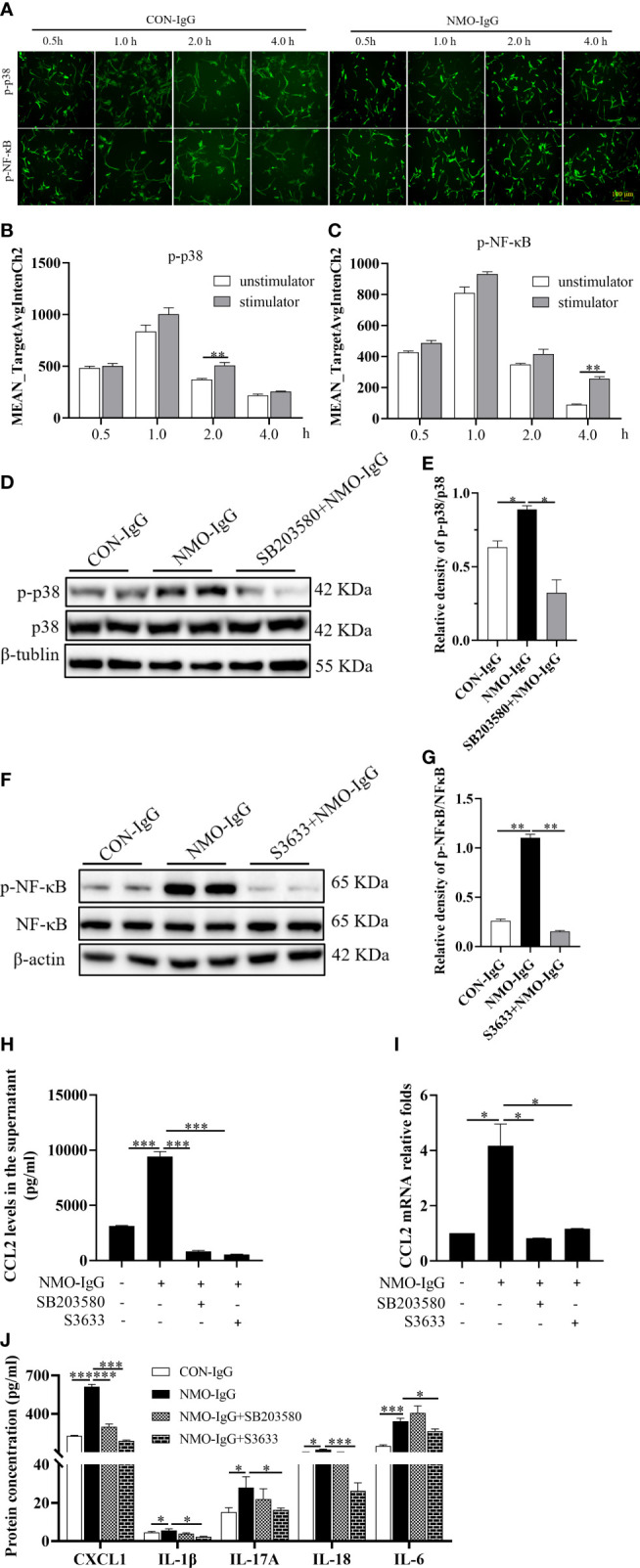
MAPK p38 and NF-κB signaling activation is involved in the CCL2 increase induced by NMO-IgG. **(A)** After cell incubation with NMO-IgG for 0.5, 1.0, 2.0, or 4.0 hours, the intracellular immunocytochemical fluorescence staining of p-p38 and p-NF-κB was analyzed using the high-content screening analyzer. Scale bar = 100 μm. **(B, C)** The fluorescence intensity of phosphorylated p38 and NF-κB was significantly increased at 2 or 4 hours after NMO-IgG stimulation (*n* =4), respectively. **(D, E)** After preincubation for 0.5 hours with the p38-selective inhibitor SB203580 (100 μM), astrocytes were stimulated with NMO-IgG, and the obvious activation of p38 signaling pathways was almost prevented by SB203580 (*n* =2). Data are presented as the mean ± SE. *p < 0.05. **(F, G)** Meanwhile, when astrocytes were pretreated for 0.5 hours with the NF-kB signaling pathway-specific inhibitor S3633 (100 mM) ahead of NMO-IgG stimulation, NF-κB phosphorylation was reversed. In addition, the release of CCL2 proteins into the supernatant **(H)** and CCL2 mRNA expression **(I)** were markedly prevented by either SB203580 or S3633. **(J)** S3633 significantly inhibited the production of CXCL1, IL-1β, IL-17A, IL-18, and IL-6, whereas SB203580 inhibited only the production of CXCL1. Data are presented as the mean ± SE of three independent experiments. ^*^ *p* < 0.05, ^**^ *p* < 0.01, and ^***^ *p* < 0.001.

To further investigate the molecular signaling pathways that mediated the increase in astrocytic CCL2 during NMO-IgG incubation, rat primary astrocytes were incubated with NMO-IgG for 0.5, 1.0, 2.0, or 4.0 hours. GFAP (red, not shown) and related inflammatory signaling pathway target proteins (green) were labeled using immunocytochemical fluorescence staining, and the acquired images are shown in **Figure 6**. The AFI of target proteins before and after NMO-IgG stimulation was analyzed by a high-content screening analyzer. Our results revealed that the AFI of phosphorylated p38 protein was significantly higher after 2 hours of NMO-IgG stimulation than before stimulation (**Figures 6A, B**), and the AFI of phosphorylated NF-κB protein was significantly higher after 4 hours of NMO-IgG stimulation (**Figures 6A, C**). These results indicate that NMO-IgG can activate the p38 and NF-κB signaling pathways. In addition, NMO-IgG can also activate the JAK1 and p42/40 signaling pathways in rat primary astrocytes (**Supplementary Figure 2**).

We then incubated cells with the p38 signaling pathway-specific inhibitor SB203580 (100 μM) and NF-κB signaling pathway-specific inhibitor S3633 (100 μM) half an hour before NMO-IgG stimulation. The Western blotting assay confirmed that NMO-IgG could activate the astrocytic p38 and NF-κB signaling pathways, and both activations could be inhibited efficiently by their corresponding inhibitors (**Figures 6D-G**). The CCL2 protein content in the supernatant and *CCL2* mRNA expression were significantly inhibited by SB203580 and S3633 (**Figures 6H, I**). Our results confirmed the involvement of p38 and NF-κB signaling pathways in NMO-IgG-stimulated CCL2 increasing derived from astrocytes.

Moreover, we found that S3633 could significantly inhibit the production of other inflammatory cytokines, including CXCL1, IL-1β, IL-17A, IL-18, and IL-6 released by NMO-IgG stimulation and significantly improve cellular inflammatory status, whereas SB203580 could only inhibit the production of CXCL1 apart from CCL2 by NMO-IgG induction (**Figure 6J**).

## 4 Discussion

The underlying pathogenic mechanisms of CNS damage in NMOSD are still not entirely understood. The most common hypothesis is that NMO-IgG produced in the periphery enters the CNS at either naturally or pathologically formed BBB sites and binds to the astrocytic surface AQP4 water channel, causing complement-dependent cytotoxicity, an inflammatory cascade response, microglia activation, myelin loss, and neuronal death (13). Although complement inhibition has a therapeutic effect in some NMO patients (14), evidence from patient pathology specimens strongly suggests that complement-mediated pathological destruction is only a secondary pathogenic mechanism of NMO. The mechanism by which AQP4 binds to astrocytes and contributes to BBB breakdown in the absence of the complement has been elucidated (7). However, the events that occur after NMO-IgG binding on astrocytes have not been elucidated. In the present study, paired samples from serum and CSF in NMOSD and OND patients were analyzed, and we found significantly higher levels of CCL2 in the CSF of NMOSD patients than in the CSF of OND patients, and the level of increase in CSF-CCL2 was AQP4-IgG seropositive specific (**Figures 1**; **2**). Based on the data obtained from patients, we deduced that CCL2 may be released immediately after the destruction begins and may play a key role in NMO-IgG damage.

We established astrocyte injury models induced by NMO-IgG *in vitro* and *in vivo*. As expected, NMO-IgG (4 U) incubation sharply increased CCL2 release in astrocytes *in vitro* (**Figure 3E**). To explore the role of CCL2, we blocked the *CCL2* gene by RNA interference or adenovirus injection and found that astrocytic CCL2 inhibition can effectively reduce NMO-IgG damage or mitigate the pathological lesions of NMO (**Figures 4**; **5**).

CCL2 is a pleiotropic chemokine that plays an important role in apoptosis, angiogenesis, secretion of cytokines and extracellular matrix proteases, and the recruitment of inflammatory cells (15–17). A large body of evidence suggests that CCL2 can promote inflammatory cell infiltration and accelerate the progression of multiple diseases (8). It has been reported that CCL2 acts on various inflammatory and immune cells (18). Furthermore, elevated phosphatase antibodies in NMOSD patients may be linked to CCL2 (19–21). CCR2-positive monocytes and T cells are attracted by CCL2. CCR2 and CCL2 have been consistently associated with a pathogenic role in BBB breakdown in patients with multiple sclerosis (22). Peripheral monocytes are known to enter the brain after traumatic brain injuries and seizures and contribute to neuronal injury and axonal damage (8, 9). Zeng et al. reported that CD14^+^CD16^++^ non-classical monocytes, which were CCR2-negative monocytes (CCR2^–^), were up-regulated in the peripheral blood of patients with NMO at the acute stage (11). Therefore, we also measured the alteration of monocyte subsets from NMOSD patients in peripheral blood at the acute or chronic phase. We found that non-classical monocytes (CD14^+^CD16^++^CCR2^–^) increased at the acute stage and classical monocytes (CD14^++^CD16^–^CCR2^+^) returned to a higher percentage at the chronic stage (**Supplementary Figure 3**). Given that CCL2 is a potent CCR2^+^ myeloid cell chemotactic stimulator and plays an important role in the progression of various CNS diseases associated with infiltration (7, 8), the CSF-CCL2 increase may account for the lower percentage of classical monocytes in blood at the acute phase. Classical monocytes in the circulation are critical for the initial inflammatory response, which can be chemotactic by CCL2 to enter the CNS, and differentiate into macrophages. The pathological features of macrophages/microglia that have invaded the focal lesions support the above explanation (10). CCL2-induced classical monocyte infiltration in the CNS can aggravate lesions, and may play an important role in the pathogenesis of NMOSD.

In the present study, we found that, in addition to CCL2, NMO-IgG was able to activate the release of multiple cytokines, such as IL-6, CXCL1, IL-1β, IL-17A, and IL-18 (**Figure 3E**). Neutralization of IL-6 can reverse the NMO-IgG-induced down-regulation of AQP4 proteins on the astrocyte surface (23). Injections of IL-1β into mice brain parenchyma were able to induce NMO-like lesions (24). The survival of astrocytes was significantly improved by blocking IL-1β and TNF-α. The sharp increase in CCL2 and other cytokines induced by NMO-IgG will disturb homeostasis and elevate the inflammatory microenvironment, possibly leading to worsening of NMO lesions. Interestingly, prevention of CCL2 expression by CCL2 siRNA interference also significantly decreases the release of five other cytokines (IL-6, IL-1β, CXCL1, IL-17A, and IL-18), suggesting that CCL2 production or release occurs ahead of production and release of the other inflammatory cytokines and that CCL2 is involved in NMO-IgG damaged initiation and expansion in astrocytes.

Although inflammatory signaling pathways were targeted by multiple factors (25, 26), we evaluated several inflammatory signaling pathways that could mediate cytokine release. As the data show, NMO-IgG was able to activate plural inflammatory signaling pathways in astrocytes, including NF-κB, MAPK p38, and JAK1. The inhibition of the NF-κB signaling pathway significantly reduced the levels of CCL2, CXCL1, IL-6, IL-1β, IL-17A, and IL-18, and effectively improved the cellular inflammatory status. Blocking the MAPK p38 signaling pathway could effectively reduce the levels of CCL2 and CXCL1 (**Figures 6H-J**).

**Figure 7:**
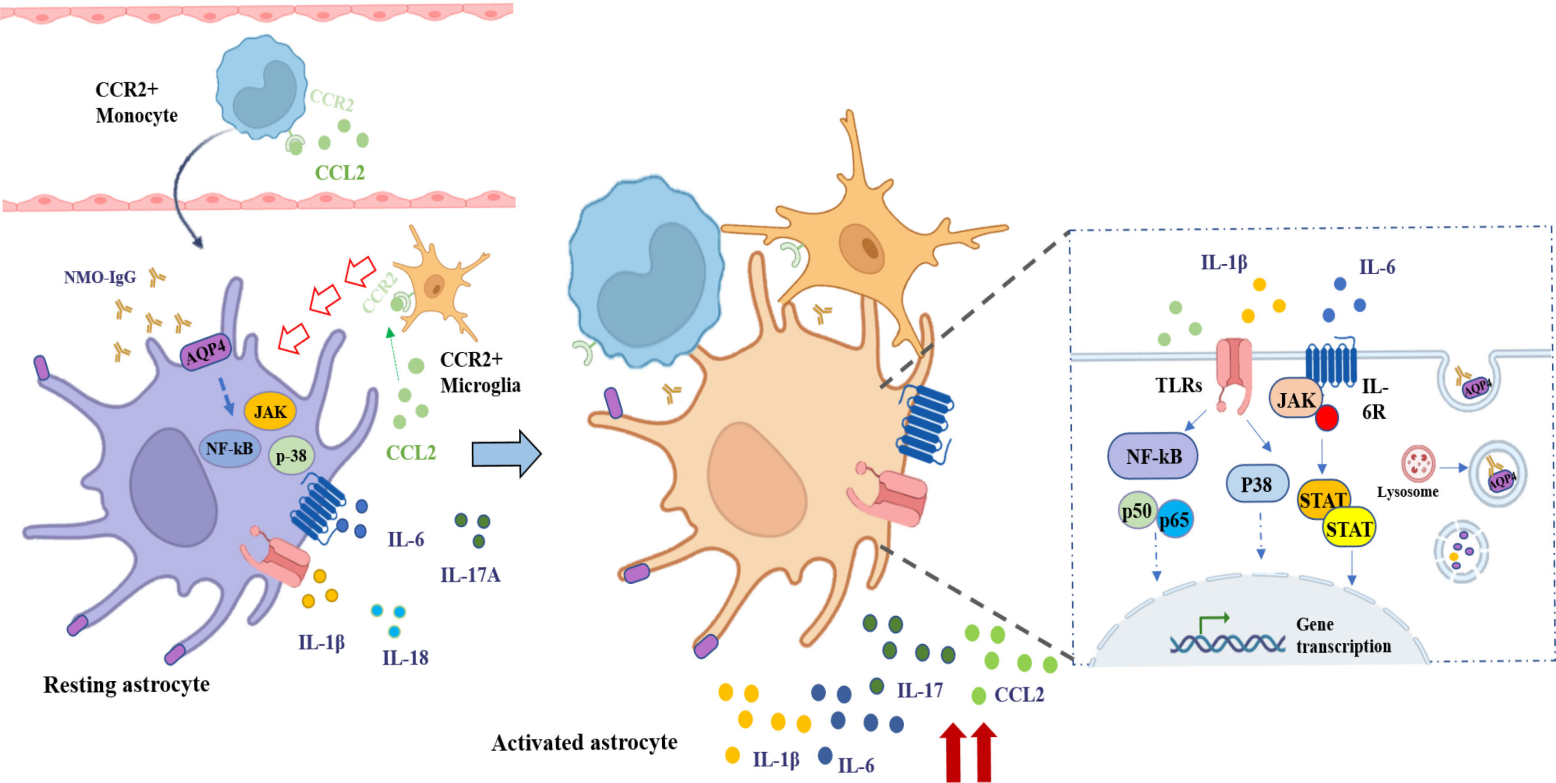
The schematic diagram shows the potential role and mechanisms of CCL2 in NMO-IgG-induced damage. The specific binding of NMO-IgG to AQP4 on the cytomembrane of astrocytes activated various intracellular inflammatory signaling pathways, for example NF-κB, p38, and JAK/STAT signaling pathways, which could induce the release of several inflammatory cytokines and chemokines, such as CCL2, IL-1β, IL-6, IL-17A, and IL-18. Subsequently, CCL2-derived peripheral CCR2-positive (CCR2^+^) monocytes penetrated the brain or attracted microglia/macrophages migrated toward the lesion focus close to astrocytes in the CNS. The interaction of CCR2^+^ cells with injured astrocytes could further stimulate the release of inflammatory cytokines, forming a positive feedback cascade loop leading to more serious damage to astrocytes.

Based on the data obtained from the present study, we drew a schematic diagram to describe the potential role and mechanisms of CCL2 in NMO-IgG-induced damage in astrocytes (**Figure 7**). The specific binding of NMO-IgG to AQP4 on the cytomembrane of astrocytes activated various intracellular inflammatory signaling pathways, for example the NF-κB, p38, and JAK/STAT signaling pathways, which would induce the release of several inflammatory cytokines and chemokines, such as CCL2, CXCL1, IL-1β, IL-6, IL-17A, and IL-18. Subsequently, CCL2-derived peripheral CCR2^+^ monocytes penetrated the brain or attracted CCR2^+^ microglia/macrophages migrated toward lesion focus close to astrocytes in the CNS. The interaction of CCR2^+^ cells with injured astrocytes would further stimulate inflammatory cytokines’ release, forming a positive feedback cascade loop that would lead to more serious damage to astrocytes. The precise molecular mechanisms underlying astrocyte pathogenesis need to be elucidated by further studies.

Some limitations of the present study need to be addressed. The OND patient cohort was small, and all serum or CSF samples were collected from a single center. We could not adequately analyze all cytokine levels after the first onset. Although RNA interference can efficiently knock down RNAs, it is prone to off-target effects. The RNA-targeting CRISPR technique could improve specificity and should be used in the future. Further studies are expected to improve and address these issues.

In conclusion, our study provides new insights into the mechanism of NMO-IgG damage to astrocytes. The role of CCL2 in NMOSD pathogenesis needs further investigation, and CCL2 may be a promising candidate target for novel drug discovery in the treatment of NMOSD.

## Data availability statement

The raw data supporting the conclusions of this article will be made available by the authors, without undue reservation.

## Ethics statement

The studies involving human participants were reviewed and approved by the Ethics Committee of Beijing Tiantan Hospital, affiliated with the Capital Medical University, Beijing, China. Written informed consent to participate in this study was provided by the participants’ legal guardian/next of kin. The animal study was reviewed and approved by the Ethics Committee of Beijing Tiantan Hospital, affiliated with Capital Medical University.

## Author contributions

All authors listed have made direct and indirect efforts. XG and LY directed the entire study and provided supervision and final checks. YPW participated in the study design and experimental performance and wrote the manuscript. JB, MY, LD, YX, HXC and HRC helped with experiments and statistical analyses. YZW, WX, HW, and XZ provided technical assistance. All authors contributed to the article and approved the submitted version.

## Supplementary material

Supplementary Figure 1 Establishment of an in vitro cell model for NMO-IgG-induced primary rat astrocyte damage.

Supplementary Figure 2 JAK1 and p42/40 signaling pathways are involved in NMO-IgG-induced damage in astrocytes.

Supplementary Figure 3 Proportion comparison of different monocyte subsets sorted by flow cytometry from NMOSD patients in the relapse or remission phase.

## References

[1] Hinson SR Lennon VA Pittock SJ. Autoimmune AQP4 channelopathies and neuromyelitis optica spectrum disorders. Handb Clin Neurol (2016) 133:377–403. doi: 10.1016/B978-0-444-63432-0.00021-9 27112688

[2] Wingerchuk DM Lennon VA Lucchinetti CF Pittock SJ Weinshenker BG. The spectrum of neuromyelitis optica. Lancet Neurol (2007) 6:805–15. doi: 10.1016/S1474-4422(07)70216-8 17706564

[3] Popescu BFG Lucchinetti CF. Pathology of demyelinating diseases. Annu Rev Pathol (2012) 7:185–217. doi: 10.1146/annurev-pathol-011811-132443 22313379

[4] Howe CL Kaptzan T Magaña SM Ayers-Ringler JR LaFrance-Corey RG Lucchinetti CF. Neuromyelitis optica IgG stimulates an immunological response in rat astrocyte cultures. Glia (2014) 62:692–708. doi: 10.1002/glia.22635 24492996 PMC5392242

[5] Du L Chang H Xu W Wei Y Wang Y Yin L. Effect of NMO-IgG on the interleukin-6 cascade in astrocytes *via* activation of the JAK/STAT3 signaling pathway. Life Sci (2020) 258:118217. doi: 10.1016/j.lfs.2020.118217 32768575

[6] Wang Y Zhang J Chang H Wang H Xu W Cong H. NMO-IgG induce interleukin-6 release *via* activation of the NF-κB signaling pathway in astrocytes. Neuroscience (2022) 496:96–104. doi: 10.1016/j.neuroscience.2022.05.038 35659638

[7] Takeshita Y Obermeier B Cotleur AC Spampinato SF Shimizu F Yamamoto E. Effects of neuromyelitis optica-IgG at the blood-brain barrier *in vitro* . Neurol(R) Neuroimmunol Neuroinflamm (2017) 4:e311. doi: 10.1212/NXI.0000000000000311 PMC5173350 28018943

[8] Varvel NH Neher JJ Bosch A Wang W Ransohoff RM Miller RJ. Infiltrating monocytes promote brain inflammation and exacerbate neuronal damage after status epilepticus. Proc Natl Acad Sci USA (2016) 113:E5665–74. doi: 10.1073/pnas.1604263113 PMC5035862 27601660

[9] Gyoneva S Kim D Katsumoto A Kokiko-Cochran ON Lamb BT Ransohoff RM. Ccr2 deletion dissociates cavity size and tau pathology after mild traumatic brain injury. J Neuroinflamm (2015) 12:228. doi: 10.1186/s12974-015-0443-0 PMC4669659 26634348

[10] Guo Y Weigand SD Popescu BF Lennon VA Parisi JE Pittock SJ. Pathogenic implications of cerebrospinal fluid barrier pathology in neuromyelitis optica. Acta Neuropathol (2017) 133:597–612. doi: 10.1007/s00401-017-1682-1 28184993 PMC5348570

[11] Zeng Q Dong X Ruan C Hu B Luo Y Luo Z. CD14(+)CD16(++) monocytes are increased in patients with NMO and are selectively suppressed by glucocorticoids therapy. J Neuroimmunol (2016) 300:1–08. doi: 10.1016/j.jneuroim.2016.09.011 27806868

[12] Hinson SR Romero MF Popescu BFG Lucchinetti CF Fryer JP Wolburg H. Molecular outcomes of neuromyelitis optica (NMO)-IgG binding to aquaporin-4 in astrocytes. Proc Natl Acad Sci U.S.A. (2012) 109:1245–50. doi: 10.1073/pnas.1109980108 PMC3268278 22128336

[13] Weinshenker BG Wingerchuk DM. Neuromyelitis spectrum disorders. Mayo Clin Proc (2017) 92:663–79. doi: 10.1016/j.mayocp.2016.12.014 28385199

[14] Pittock SJ Lennon VA McKeon A Mandrekar J Weinshenker BG Lucchinetti CF. Eculizumab in AQP4-IgG-positive relapsing neuromyelitis optica spectrum disorders: an open-label pilot study. Lancet Neurol (2013) 12:554–62. doi: 10.1016/S1474-4422(13)70076-0 23623397

[15] Fantuzzi L Tagliamonte M Gauzzi MC Lopalco L. Dual CCR5/CCR2 targeting: opportunities for the cure of complex disorders. Cell Mol Life Sci (2019) 76:4869–86. doi: 10.1007/s00018-019-03255-6 PMC6892368 31377844

[16] Zhou Y Tang H Liu J Dong J Xiong H. Chemokine CCL2 modulation of neuronal excitability and synaptic transmission in rat hippocampal slices. J Neurochem (2011) 116:406–14. doi: 10.1111/j.1471-4159.2010.07121.x PMC3018532 21105875

[17] Zhang K Luo J. Role of MCP-1 and CCR2 in alcohol neurotoxicity. Pharmacol Res (2019) 139:360–66. doi: 10.1016/j.phrs.2018.11.030 PMC6360095 30472461

[18] Karpus WJ Lukacs NW Kennedy KJ Smith WS Hurst SD Barrett TA. Differential CC chemokine-induced enhancement of T helper cell cytokine production. J Immunol (1997) 158:4129–36. 9126972

[19] Pan J Zhou L Zhang C Xu Q Sun Y. Targeting protein phosphatases for the treatment of inflammation-related diseases: From signaling to therapy. Signal Transduct Target Ther (2022) 7:177. doi: 10.1038/s41392-022-01038-3 35665742 PMC9166240

[20] Slavin YN Bo M Caggiu E Sechi G Arru G Bach H. High levels of antibodies against PtpA and PknG secreted by mycobacterium avium ssp. paratuberculosis are present in neuromyelitis optica spectrum disorder and multiple sclerosis patients. J Neuroimmunol (2018) 323:49–52. doi: 10.1016/j.jneuroim.2018.07.007 30196833

[21] Lee JH Kim H Woo JH Joe E Jou I. 5, 8, 11, 14-eicosatetraynoic acid suppresses CCL2/MCP-1 expression in IFN-γ-stimulated astrocytes by increasing MAPK phosphatase-1 mRNA stability. J Neuroinflamm (2012) 9:34. doi: 10.1186/1742-2094-9-34 PMC3308915 22339770

[22] Mahad D Callahan MK Williams KA Ubogu EE Kivisäkk P Tucky B. Modulating CCR2 and CCL2 at the blood-brain barrier: Relevance for multiple sclerosis pathogenesis. Brain J Neurol (2006) 129:212–23. doi: 10.1093/brain/awh655 16230319

[23] Haruki H Sano Y Shimizu F Omoto M Tasaki A Oishi M. NMO sera down-regulate AQP4 in human astrocyte and induce cytotoxicity independent of complement. J Neurol Sci (2013) 331:136–44. doi: 10.1016/j.jns.2013.05.035 23809190

[24] Kitic M Hochmeister S Wimmer I Bauer J Misu T Mader S. Intrastriatal injection of interleukin-1 beta triggers the formation of neuromyelitis optica-like lesions in NMO-IgG seropositive rats. Acta Neuropathol Commun (2013) 1:5. doi: 10.1186/2051-5960-1-5 24252536 PMC3776214

[25] Zeng L Jiang H Ashraf GM Liu J Wang L Zhao K. Implications of miR-148a-3p/p35/PTEN signaling in tau hyperphosphorylation and autoregulatory feedforward of Akt/CREB in alzheimer's disease. Mol Ther Nucleic Acids (2022) 27:256–75. doi: 10.1016/j.omtn.2021.11.019 PMC8714918 35024240

[26] Ma H Wang L Lv W Lv Z. Effects of miR-7 on hcy-induced rat cerebral arterial vascular smooth muscle cell proliferation, migration and inflammatory factor expression by targeting MMP-14 to regulate TLR4/NF-κB signaling pathway. Cell Mol Biol (2020) 66:12–7. 33287916

