# Supplementary figures and images for: Targeting chemoattractant chemokine (C–C motif) ligand 2 derived from astrocytes is a promising therapeutic approach in the treatment of neuromyelitis optica spectrum disorders
Source: Front Immunol. 2023 Mar 28;14:1144532. doi: 10.3389/fimmu.2023.1144532 (PMC10086366; doi:10.3389/fimmu.2023.1144532)

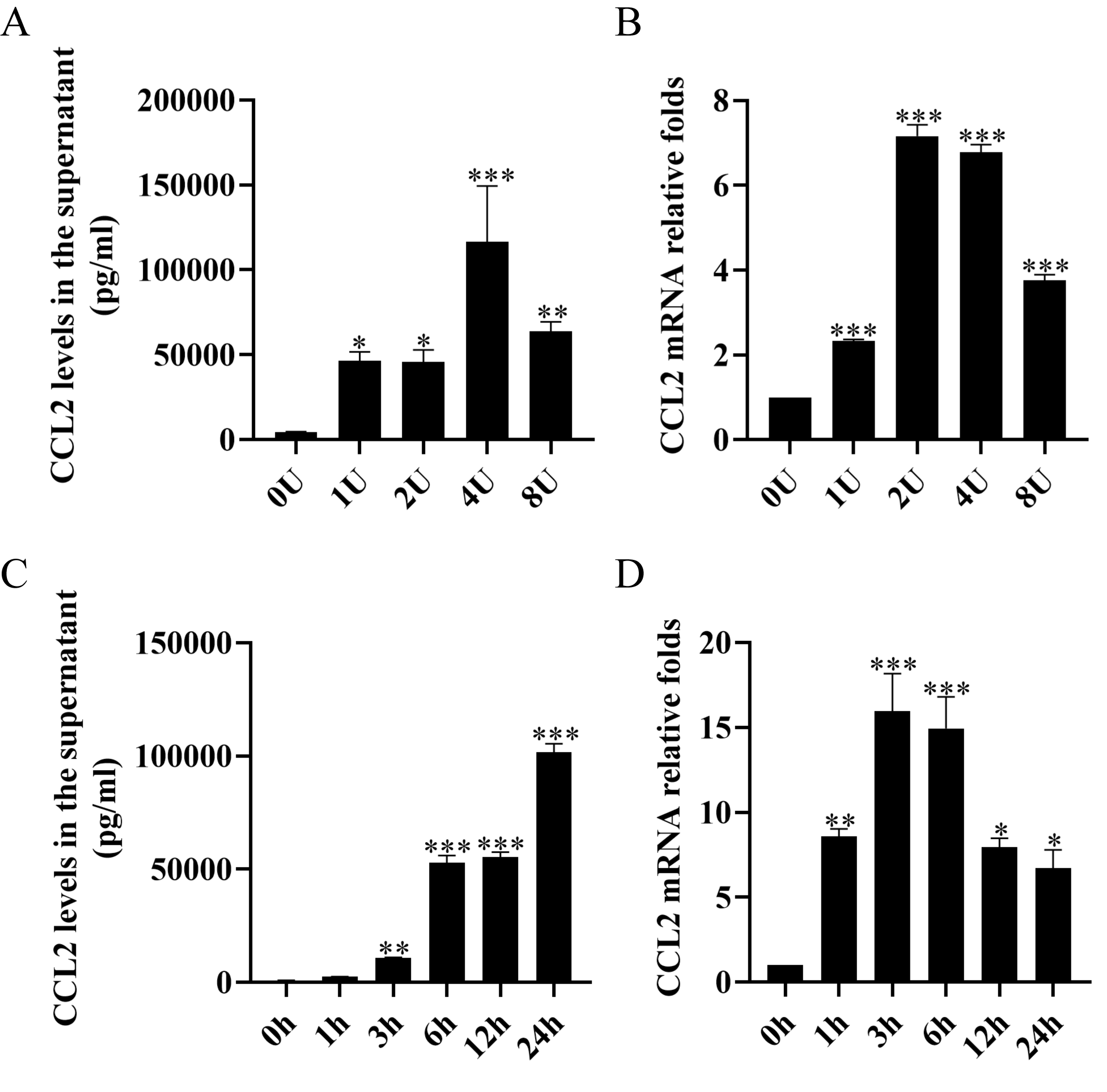

Supplement: Supplementary Figure 1 — Establishment of an in vitro cell model for NMO-IgG-induced primary rat astrocyte damage. [file Image_1.tif]

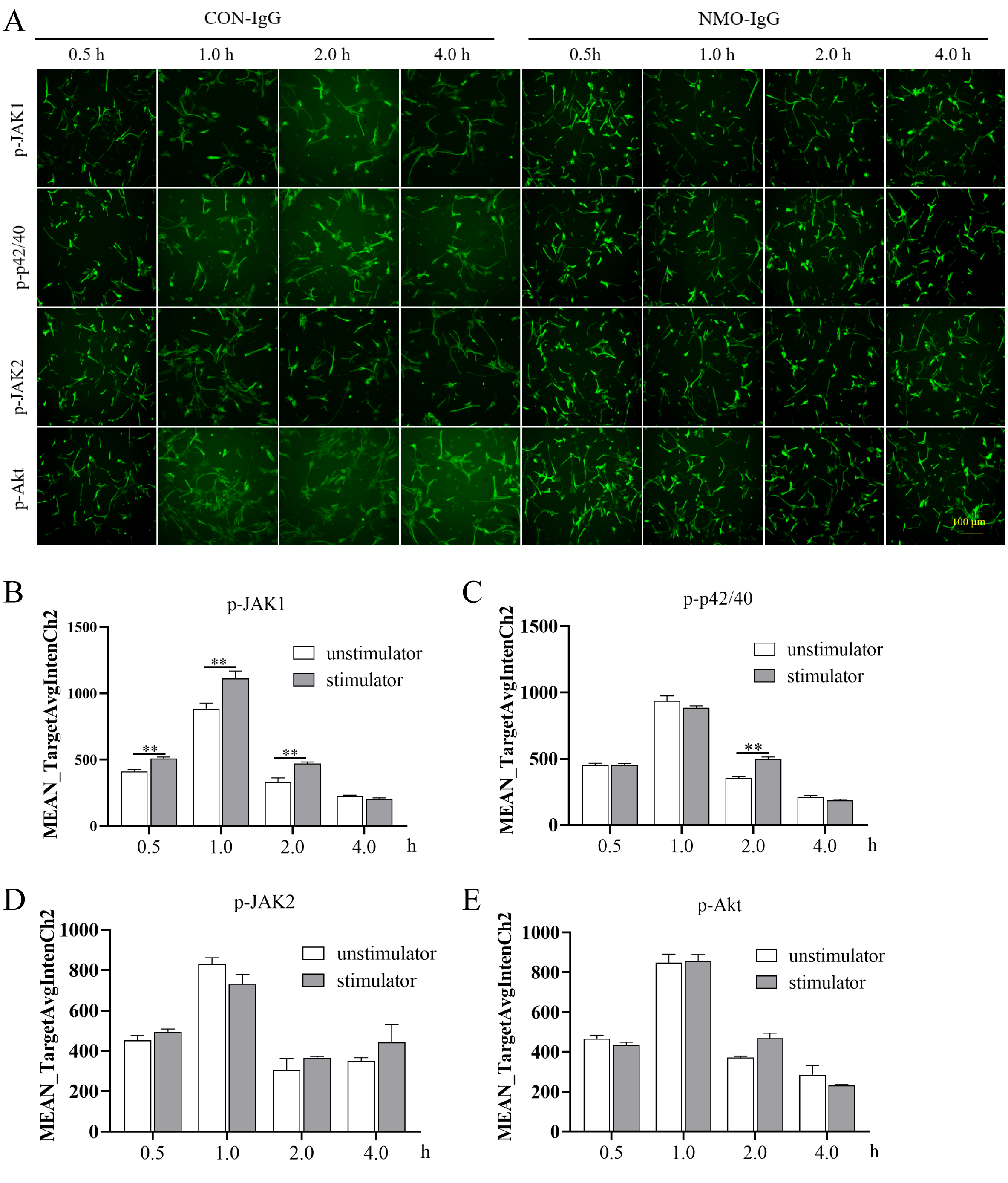

Supplement: Supplementary Figure 2 — JAK1 and p42/40 signaling pathways are involved in NMO-IgG-induced damage in astrocytes. [file Image_2.tif]

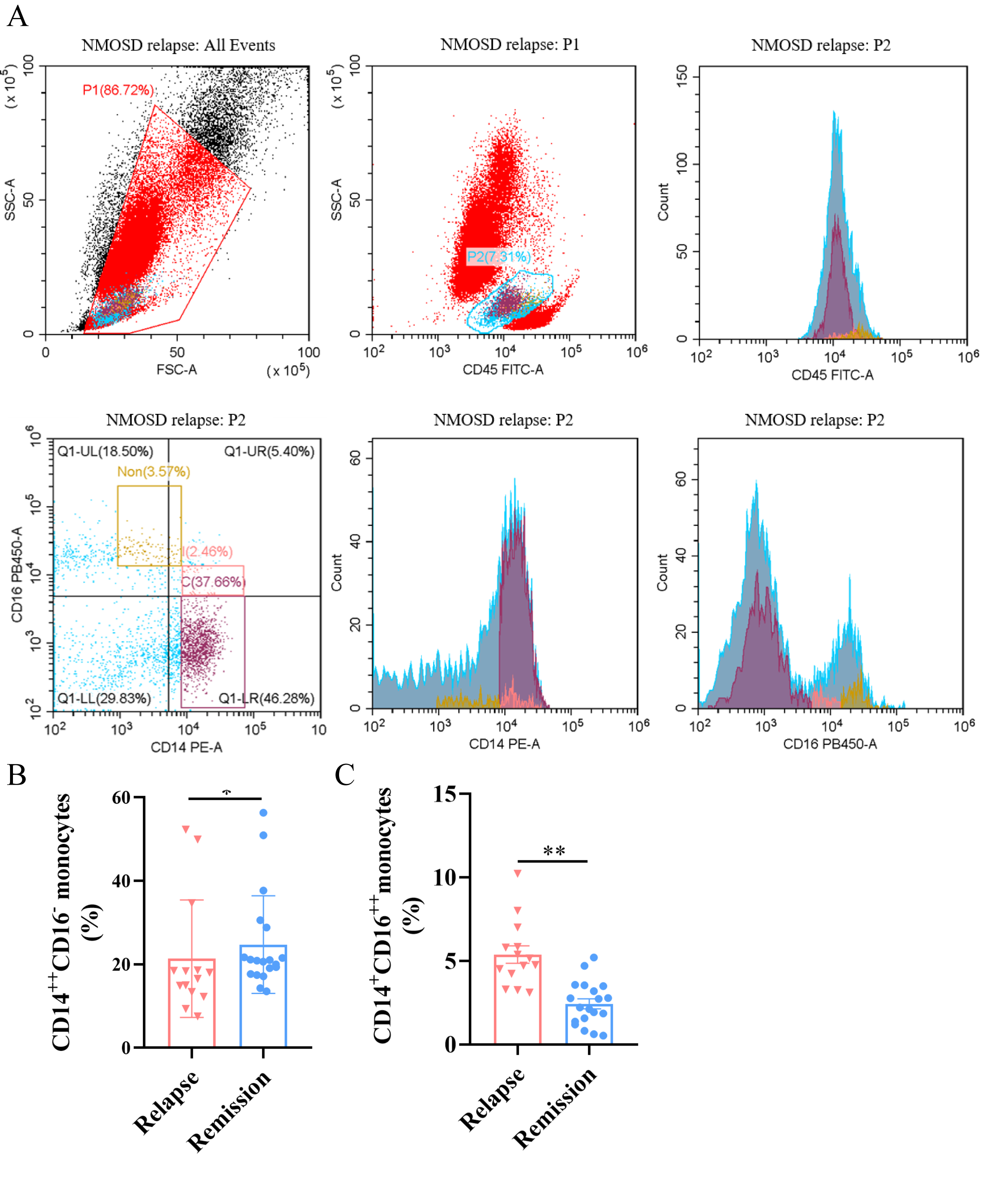

Supplement: Supplementary Figure 3 — Proportion comparison of different monocyte subsets sorted by flow cytometry from NMOSD patients in the relapse or remission phase. [file Image_3.tif]
